# Virtual Travel Training for Autism Spectrum Disorder: Proof-of-Concept Interventional Study

**DOI:** 10.2196/games.8428

**Published:** 2018-03-20

**Authors:** Marco Simões, Miguel Bernardes, Fernando Barros, Miguel Castelo-Branco

**Affiliations:** ^1^ Coimbra Institute for Biomedical Imaging and Translational Research Institute of Nuclear Sciences Applied to Health University of Coimbra Coimbra Portugal; ^2^ Center for Informatics and Systems University of Coimbra Coimbra Portugal; ^3^ Faculty of Medicine University of Coimbra Coimbra Portugal

**Keywords:** Autism Spectrum Disorder, serious games, virtual reality, virtual reality therapy, travel train, bus.

## Abstract

**Background:**

Autism Spectrum Disorder (ASD) is a neurodevelopmental disorder characterized by impairments in social interaction and repetitive patterns of behavior, which can lead to deficits in adaptive behavior. In this study, a serious game was developed to train individuals with ASD for an important type of outdoor activity, which is the use of buses as a means of transportation.

**Objective:**

The aim of this study was to develop a serious game that defines a “safe environment” where the players became familiar with the process of taking a bus and to validate if it could be used effectively to teach bus-taking routines and adaptive procedures to individuals with ASD.

**Methods:**

In the game, players were placed in a three-dimensional city and were submitted to a set of tasks that involved taking buses to reach specific destinations. Participants with ASD (n=10) underwent between 1 to 3 training sessions. Participants with typical development (n=10) were also included in this study for comparison purposes and received 1 control session.

**Results:**

We found a statistically significant increase in the measures of knowledge of the process of riding a bus, a reduction in the electrodermal activity (a metric of anxiety) measured inside the bus environments, and a high success rate of their application within the game (93.8%).

**Conclusions:**

The developed game proved to be potentially useful in the context of emerging immersive virtual reality technologies, of which use in therapies and serious games is still in its infancy. Our findings suggest that serious games, using these technologies, can be used effectively in helping people with ASD become more independent in outdoor activities, specifically regarding the use of buses for transportation.

## Introduction

Autism Spectrum Disorder (ASD) is a neurodevelopmental disorder responsible for impairments in social communication and interaction and restricted repetitive patterns of behavior, interests, or activities. Current ASD prevalence is estimated to be around 1% of the population, worldwide [1]. Given that a cure is yet to be found, individuals rely on interventional approaches to improve and overcome their impairments. Further increasing the relevance of rehabilitation driven strategies is the fact that still only a minority of individuals with ASD are able to live independently in adulthood [1].

Virtual reality (VR) consists of artificial, 3D, computer-generated environments which the user can explore and interact with [2]. VR usually takes advantage of different types of devices such as head-mounted displays and controllers to deliver the stimulation and provide means of interaction that lead to immersive experiences [3]. The immersiveness of an experience is measured by the level of fidelity, concerning all sensory modalities, that a VR system can provide. Thus, immersion is objective, measurable, and depends only on the technology used by the VR system. Presence, on the other hand, is the human reaction to immersion, the feeling of actually being in the virtual environment and behaving as such [4,5,6]. In fact, the immersive VR technologies available nowadays are able to present users with experiences realistic enough to trick the mind and create a feeling of presence within the environment [7]. These technologies have already been used and proven effective in therapies for posttraumatic stress disorder [8], phobias [9], as well as ASD [10]. There are several reasons that justify the use of VR in those approaches. Its capacity to provide safe, realistic, and controlled environments [11] make therapy possible for people who, due to physical (eg paralysis) or psychological (eg, anxiety) reasons, cannot undergo exposure in real life situations. Moreover, this creates the possibility of applying therapies at ease without the need to go to a specific location where real exposure occurs. Finally, even when using low-budget hardware and software, it has been proven that VR therapies can be as effective as exposure in real life [9].

Serious games have also been proven effective in ASD therapies, not only for including game design techniques to keep the players motivated, but also because individuals with ASD are often interested in computer-based activities [12]. However, according to Zakari et al [13], most of the serious games developed for ASD rehabilitation between 2004 and 2014 are delivered through nonimmersive VR (eg, desktops, tablets) and focus mainly on communication and social skill development [13]. This highlights both the importance of focusing on other relevant ASD impairments, including executive function in outdoor situations, as well as exploring the potential of these new immersive technologies.

The aim of this study was to develop and validate a serious game which uses a VR headset (Oculus Rift) as a proof of concept for rehabilitation of individuals with ASD. Teaching a person with ASD to use public transportation requires parents or therapists to practice with them until they are ready and comfortable enough to perform this task alone (basically, the same process used with people with typical development, but with all the obstacles set by ASD particularities). In fact, being able to engage in outdoor activities such as the efficient use of public transport can be particularly challenging for people with ASD due to deficits in adaptive behavior [14] and anxiety [15]. This project intended to ease this process by creating and validating a game that prepares the players to use, in this case, buses for transportation. This included not only teaching the required skills, but also making them comfortable with the involved procedures and environments. To our knowledge, this is the first study to use VR training for teaching the process of bus-taking for people with ASD.

## Methods

In this section, we describe the game, the experimental setup, the participants, and the validation procedure.

### Game Description

**Figure 1:**
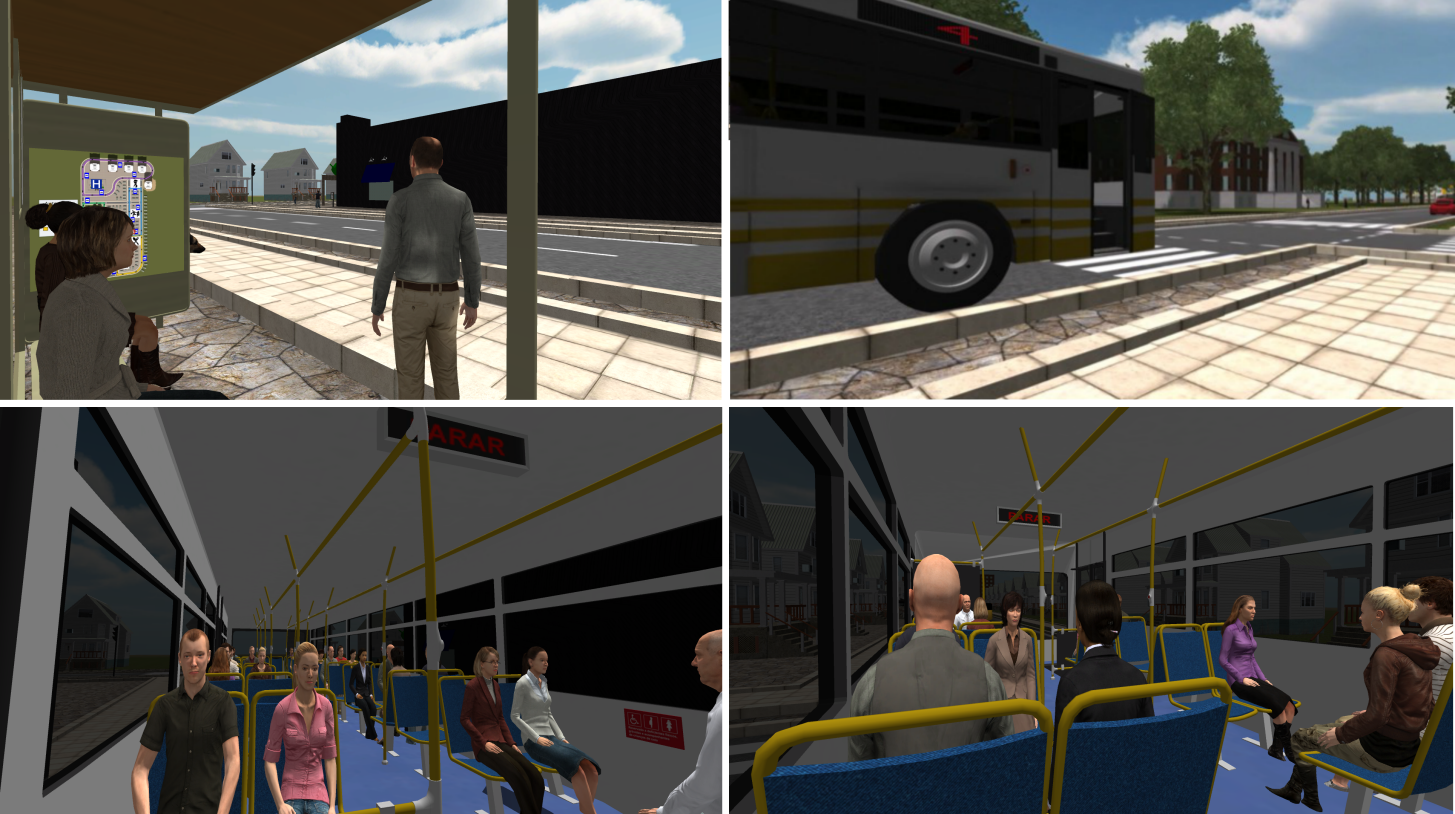
Screenshot from the virtual environment, showing two views from the bus stop perspective on the top, and two views from inside the bus on the bottom. On the top left corner, we can see one bus stop, with other people waiting for the bus, and the map to be used by the participant on the wall. On the top right, we see a bus with its designated number signaled in red, and some traffic on the street. The bottom images show two perspectives from inside of the bus.

The game was developed in-house with the aim of preparing individuals with ASD to use buses for transportation. To achieve that, it places the user in a three-dimensional city and sets a task that is completed by riding buses to reach a specific destination. Figure 1 shows different screenshots of the city and the buses.

**Figure 2:**
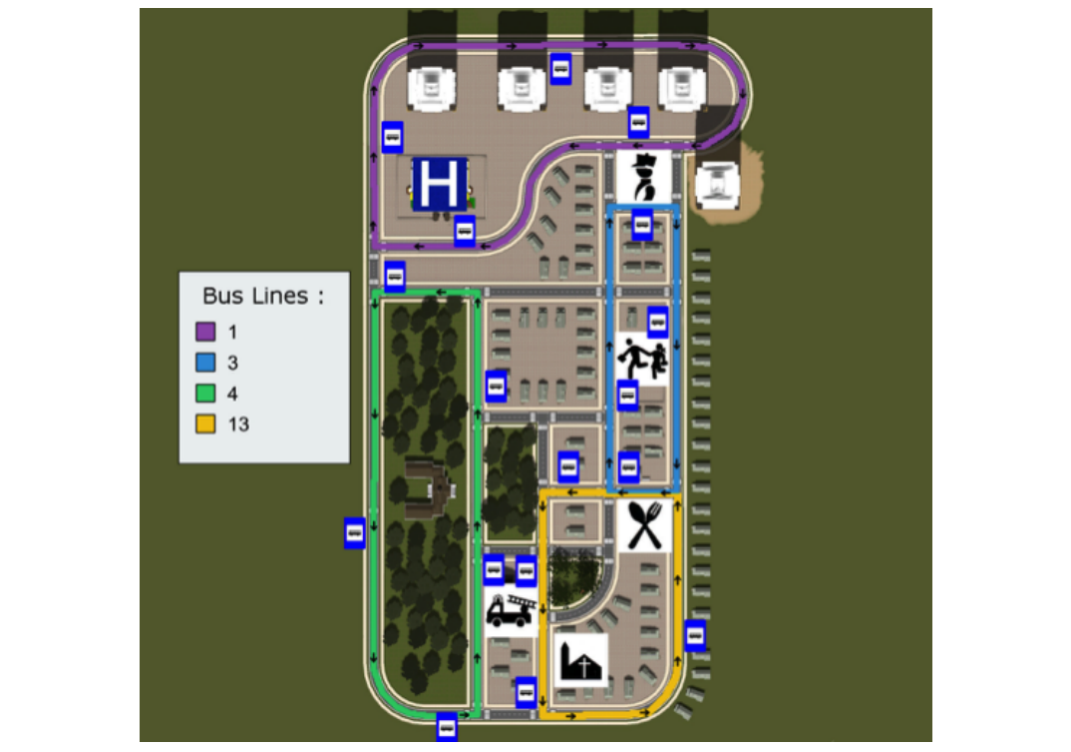
City map showing the bus lines, stops, and important places like the hospital, church, restaurant, and others.

There are several different buses driving in 4 pre-defined routes within the city. Figure 2 shows the map of the city with the 4 bus lines available. The player can enter any of these buses, validate his/her ticket, choose a place to sit, and press the STOP button, requesting the bus to stop, and leave the bus. Before starting the game, it is possible to choose from 7 different tasks, 4 of them labelled by complexity as simple (the player only needs to take 1 bus to reach the destination) and 3 labelled as complex (the player needs to take 2 buses to reach the destination). Each task has 2 levels of difficulty: an easy and a hard mode. The easy mode leads the player, step by step, to the destination, while in the hard mode, the player is only told the place he/she must go to. At the end of each task, a scoring system evaluates the performance of the player on 2 different components: “Actions” (the capacity of the player to memorize and execute bus norms, eg, validating the ticket or sitting on unreserved seats) and “Route” (the capacity of the player to plan a route to the destination, eg, if the player took the right buses in the right bus stops).

**Figure 3:**
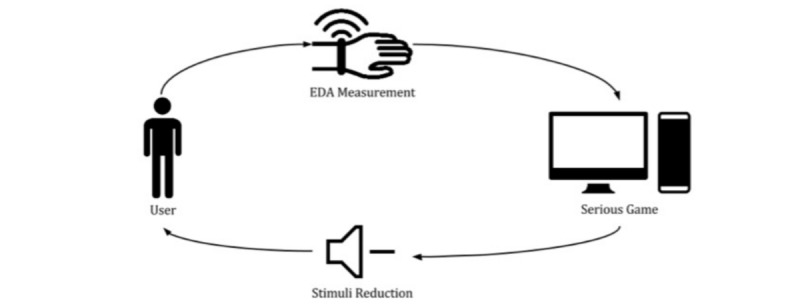
Biofeedback loop diagram. The level of electrodermal activity is measured from the participant by the game. If it detects a peak of activity, it decreases the level of stimuli and noise in the scene.

The game includes several objects/elements, such as people, traffic, and dogs, which might cause anxiety, depending on the player. For that reason, a biofeedback system was implemented to ensure that, while the player becomes familiarized with the environment, it never becomes hostile to him/her. This is achieved by assessing the anxiety felt by the player through the analysis of the electrodermal activity (EDA) and reducing the stimulus clutter the player is exposed to, in real time, in case high anxiety levels are reached (eg, reducing the amount of noise in the environment). Figure 3 schematizes the biofeedback loop.

### Recruitment

For this project, 2 groups were selected: a clinical and a control group. For the clinical group, 10 participants with ASD, whose diagnosis followed the criteria established on the Diagnostic and Statistical Manual of Mental Disorders, 5^th^ Edition [1], were recruited from Associação Portuguesa para as Perturbações do Desenvolvimento e Autismo de Viseu (APPDA-Viseu). For the clinical group, 9 males and 1 female were recruited, with a mean age of 18.8 (SD 4.5) years. In the same group, 8 were without intellectual disability (IQ equal to 70 or higher) and 2 were with mild intellectual disability (IQ between 50 and 69 [16]). For the control group, 10 individuals with typical development (TD) were also recruited; 4 males and 6 females, with a mean age of 21.9 (SD 3.56) years. The groups were matched by age (t(18)=−1.633, *P*=.12). Participants gave oral consent, and a written informed consent was obtained from their parents/guardians or themselves if they were adults with sufficient autonomy.

### Intervention Protocol

The ASD group underwent an intervention of 3 sessions of increasing complexity and difficulty (see Table 1), with a duration between 20 and 40 minutes each. Of the 10 participants recruited with ASD, only 6 performed all the intervention sessions of the study due to scheduling issues. During the recruitment, participants completed a questionnaire to assess their experience in using buses for transportation. With an exception for 1 of the participants, all the participants were unable to take buses autonomously at the beginning of the study.

The control group was submitted to a single session (corresponding to the first of the patients). This group was used to provide a task baseline and to assess the capacity of the serious game to identify differences between groups.

The sessions took place in APPDA-Viseu for the ASD participants and in the Institute of Biomedical Imaging and Life Sciences for the TD participants. In all of them, the same research staff was present. This study and all of the procedures were reviewed and approved by the Ethics Commission of our Faculty of Medicine from University of Coimbra and were conducted in accordance with the declaration of Helsinki.

### Session Procedure

In each session, the players received a tutorial (to learn or review the game controls) and a task. The task difficulty and complexity changed from session to session, as shown in Table 1. At the end of every session, participants were asked to describe the process of riding a bus, from the moment they arrived at the bus stop, until they reached their destination, but never received feedback on the answer given. Their responses were recorded in a checklist containing all the steps existing in a bus trip (Textbox 1).

### Acquisition Setup

**Figure 4:**
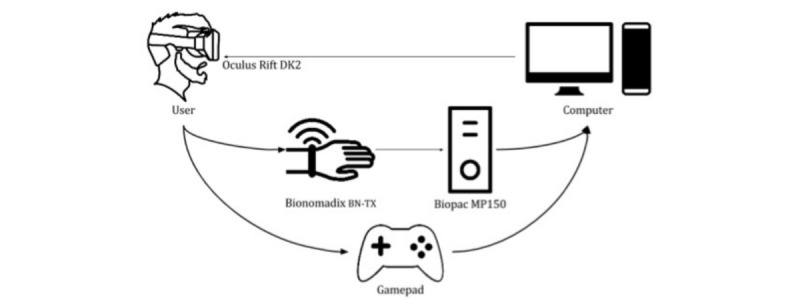
Diagram of the setup used during the sessions, including the virtual reality headset, game controller, biosignal recorder, and the main computer.

In every session, players sat on a swivel chair and wore a bracelet for wireless EDA recording (Biopac Bionomadix BN-PPGED and MP150 amplifier). All tasks were run on a laptop computer (Windows 8.1, 16.0 GB RAM and an IntelCore i7 2.50 GHz processor). The head-mounted display used was Oculus Rift Development Kit 2, firmware version 2.12, and a gamepad was used for input. Three participants with ASD and 1 with TD received the tasks without the Oculus Rift, due to vision impairments. Figure 4 illustrates the setup used.

**Table 1:** Task complexity and difficulty per session.

Metric	Session 1	Session 2	Session 3
Difficulty	Easy	Hard	Hard
Complexity	Simple	Simple	Complex

**Checklist with steps existing in a bus trip.**

- Wait for the bus
- Enter in the bus
- Validate ticket
- Avoid reserved seats
- Sit
- Wait until getting close to the destination
- Press stop button
- Leave the bus

### Metrics and Outcome Measures

Because the main objective of the serious game was to teach how to take the bus, we defined two main outcome measures to evaluate the knowledge of the process of riding the bus. One is measured automatically by the game and the other is measured using the debriefing. Both consist of the percentage of the checklist (Textbox 1) performed correctly.

#### Actions Accuracy

The game identified every action of the participant (entering the bus, ticket validation, etc) and calculated the actions accuracy based on the equation: number of correct actions/number of expected actions (Textbox 1).

#### Debriefing Accuracy

After the game, we asked the participants to describe the step-by-step process of riding a bus and calculated an accuracy based on the same equation of the actions accuracy.

#### Additional Metrics

##### Task Duration

Duration, in minutes, of the time taken to complete the task in each session. Since the tasks changes in complexity and difficulty, this metric is not directly comparable between sessions. Nevertheless, it is useful to analyze intersubject variability and to perform intergroup comparisons for the first session.

##### Anxiety Level

The variation of EDA values during the session was used as a measure of anxiety. To calculate it, we detrended the signal, subtracting it to its best fit to a straight-line (for details, see detrend implementation in MathWorks Matlab), then calculated the standard deviations of the detrended EDA values. We then created heat maps of anxiety peaks in the “virtual city”, where it is possible to highlight the game locations where EDA peaks occurred, corresponding to anxiety events felt by the players. If the locations of anxiety peaks are the same between subjects and sessions, those locations will become red, but if they are sparse, their representations are green and blue. The heat maps were created using heatmap.js [17], an open source heat map visualization library for JavaScript. Since the game has two major different situations (the city streets and the inside of the buses), two different conditions were defined: one representing the anxiety felt on the streets of the city and another with the anxiety felt inside the buses.

### Statistical Analysis

For every metric, normality was assessed using histograms, Q-Q plots and the Kolmogorov-Smirnov test. Normality test results were used to choose between parametric and nonparametric test, accordingly.

#### Clinical vs Control Group Analysis:

The metrics actions accuracy, debriefing accuracy, and task duration were not normally distributed. Therefore, we used the Mann-Whitney U test for the between-group comparison of those metrics. Anxiety Level data was nevertheless normally distributed. Thus, we used two-sample *t* test for those between-groups comparisons.

### Intervention Analysis (Intragroup Analysis for the Clinical Group):

As expected from the intergroup analysis, Actions Accuracy, Debriefing Accuracy, and Task Duration were not normally distributed. Paired data for last and first session were therefore compared using a Wilcoxon Sign-rank test. Regarding Anxiety Level, a one-sample *t* test was used for the paired data (last and first session). Additionally, we generated heat maps of locations where consistent peaks of anxiety where identified.

## Results

The results are organized in two sections, one for the intergroup analysis and another for the within-subject analysis of the clinical group. In the figures presented, the error bars always represent the standard error of the mean, and the horizontal bar represents the median when considering nonparametric data (actions and debriefing accuracies, as well as task durations) and the mean when considering parametric data (EDA measures).

### Clinical vs Control Group Results

**Figure 5:**
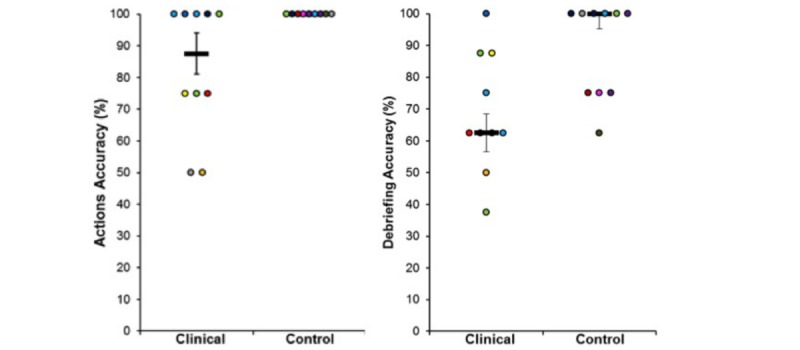
Left: Actions accuracy for both groups in the first session. The control group (typical development) had a perfect performance, while participants in the clinical group missed some actions. Right: Debriefing accuracy for both groups. Higher, but not perfect, accuracy was found in the control group.

Regarding the actions accuracy (percentage of correct actions performed during the task), we found a statistically significant difference between groups. The Mann-Whitney U test indicated that the actions accuracy of the control group (median=100%) was greater than for the clinical group (median=88%), U=75.0, *P*=.01. When analyzing the knowledge retained by the debriefing, the control group (median=100%) has greater accuracy than the clinical group (median=62.5%), as shown by the Mann-Whitney U test, U=80.5, *P*=.02 (Figure 5).

**Figure 6:**
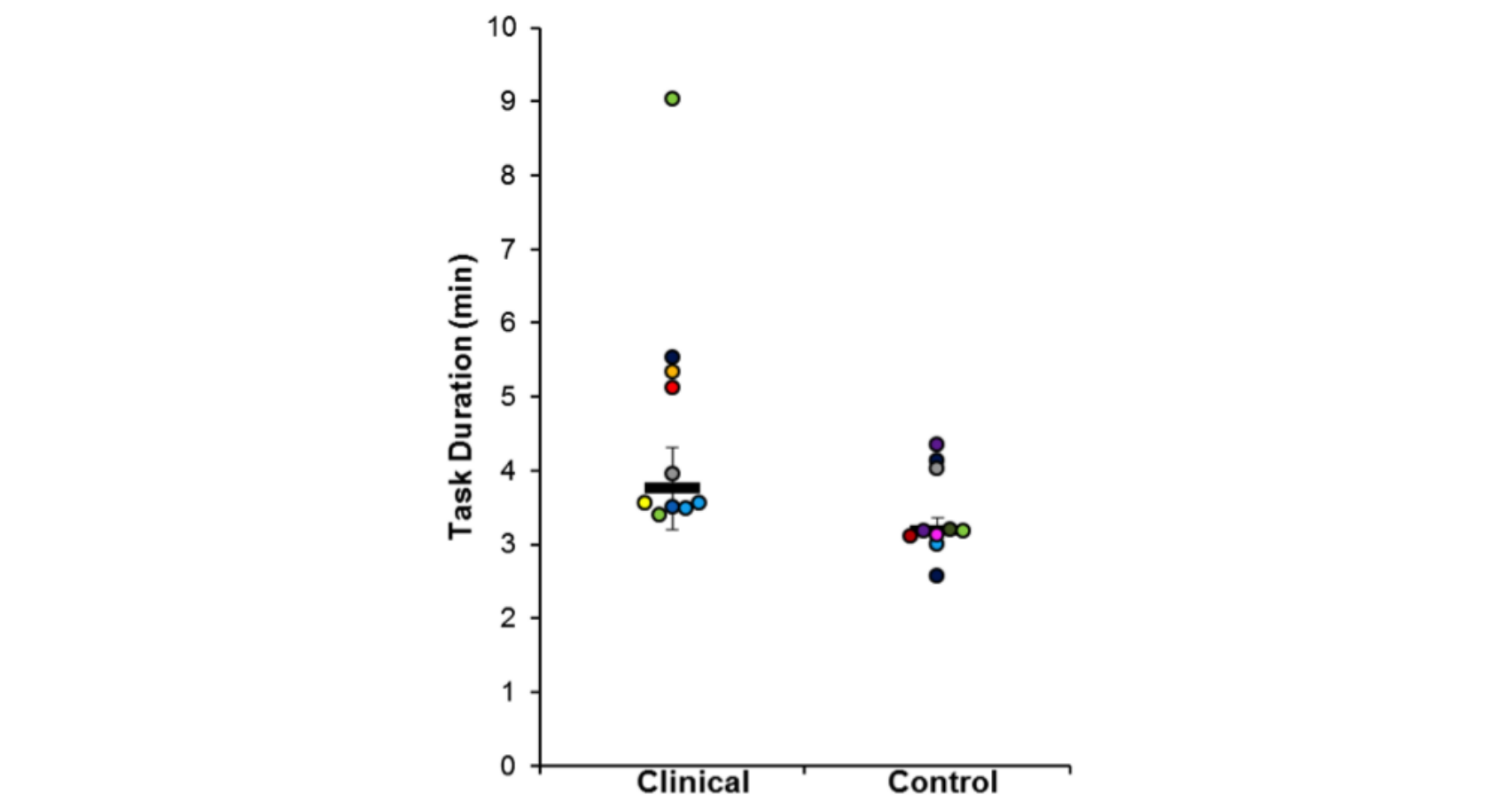
Task duration for session 1 from each group.

In terms of Task Duration, Figure 6 shows the between-group differences. A Mann-Whitney U test showed that the task duration of the ASD group (median=3.76) was statistically significant higher than for the control group (median=3.19), U=18.0, *P*=.02.

**Figure 7:**
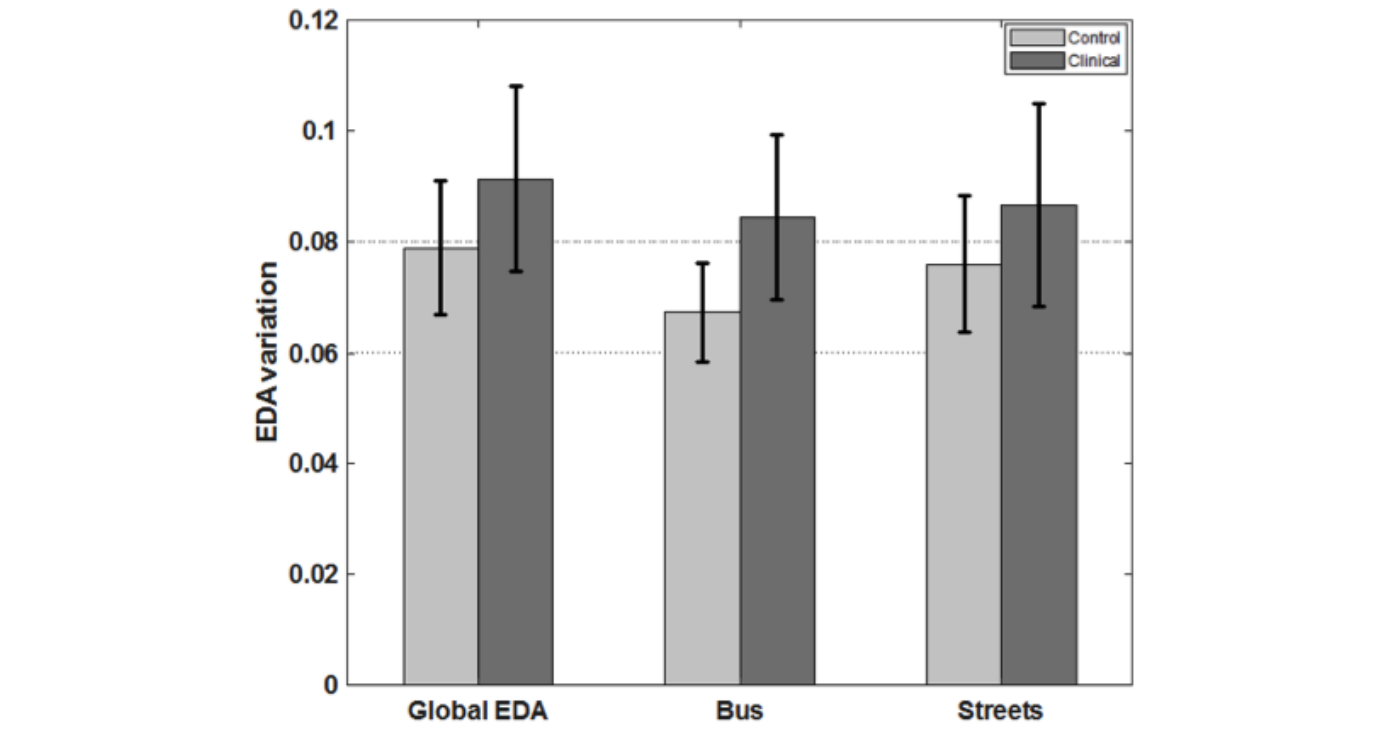
Anxiety levels for each group (mean and standard error) for the overall task and two subconditions: inside the bus and outside (streets). The clinical group presents higher values for all the settings, although without statistical significance. EDA: electrodermal activity.

Regarding the anxiety level of each group, we see a trend for higher values for the clinical group for all the scenarios (global EDA, inside the bus and outdoors, in the street). Figure 7 shows the mean EDA fluctuations of each group in each condition. However, two-sample *t* tests showed no statistically significant differences (possibly because of the biofeedback implementation) for either of the conditions (global EDA: t(18)=−0.60, *P*=.56; bus condition: t(18)=−0.99, *P*=.33; streets condition: t(18)=−0.48, *P=*.63).

### Intervention Results

**Figure 8:**
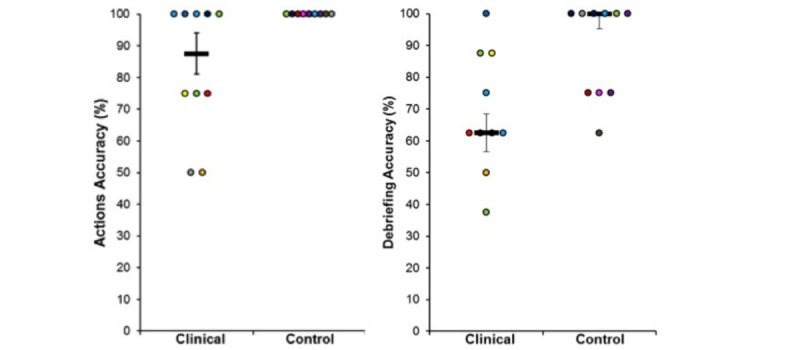
Actions accuracy for the clinical group, measured “inside the game” throughout the intervention sessions.

**Figure 9:**
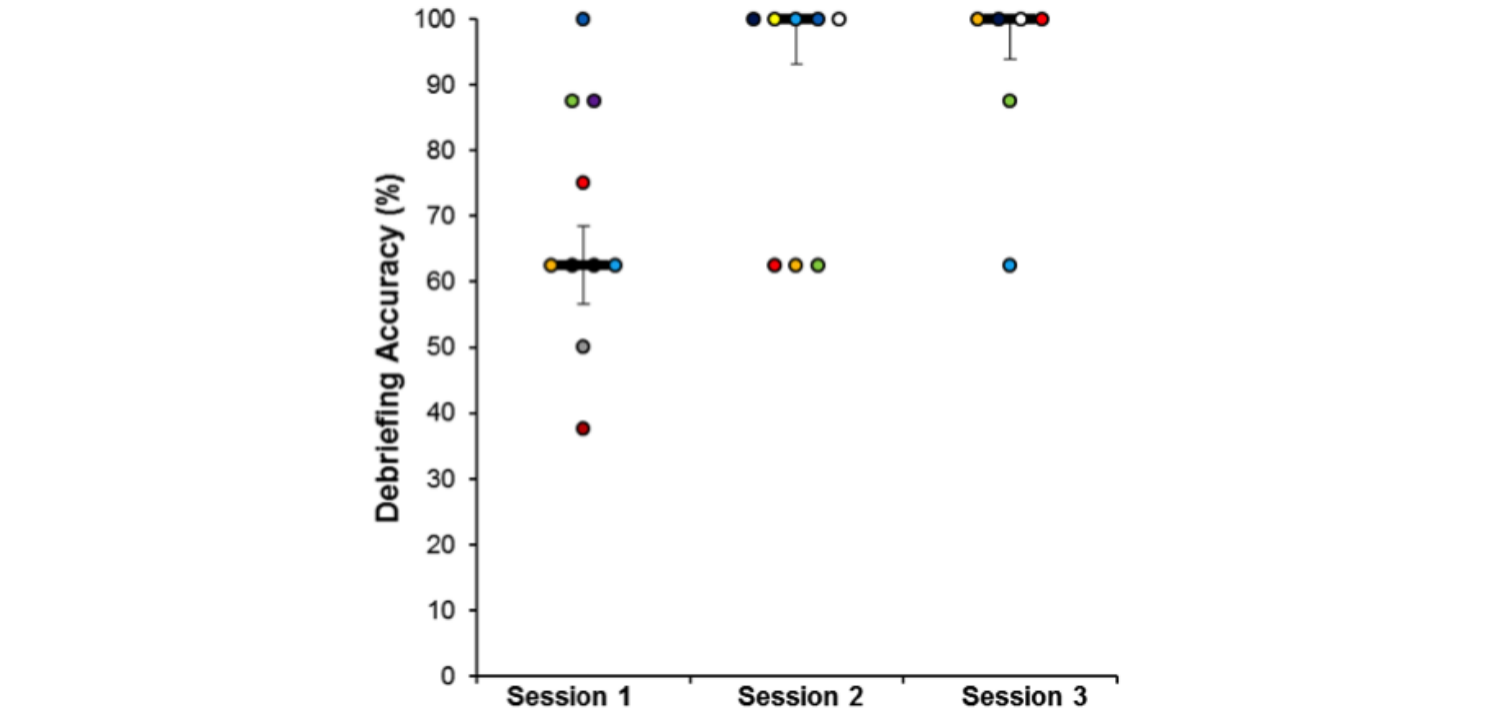
Debriefing accuracy of the intervention group for each session.

We then compared the main outcome measures pre- and postintervention for the 6 participants who completed the 3 sessions. The in-game measure (actions accuracy) evolved positively throughout the sessions (preintervention median accuracy=75.0%, postintervention median accuracy=93.8%; see Figure 8), with a Wilcoxon signed-rank test showing a small trend for significant differences (Z=1.63, *P*=.10). Even though the differences were not statistically significant when using “in-game” measures, the same test applied to the Debriefing Accuracy show a significant increase (Z=2.22, *P*=.03) from session 1 (median=68.8%) to session 3 (median=100.0%). Figure 9 illustrates the evolution of the debriefing checklist accuracy throughout the sessions.

**Figure 10:**
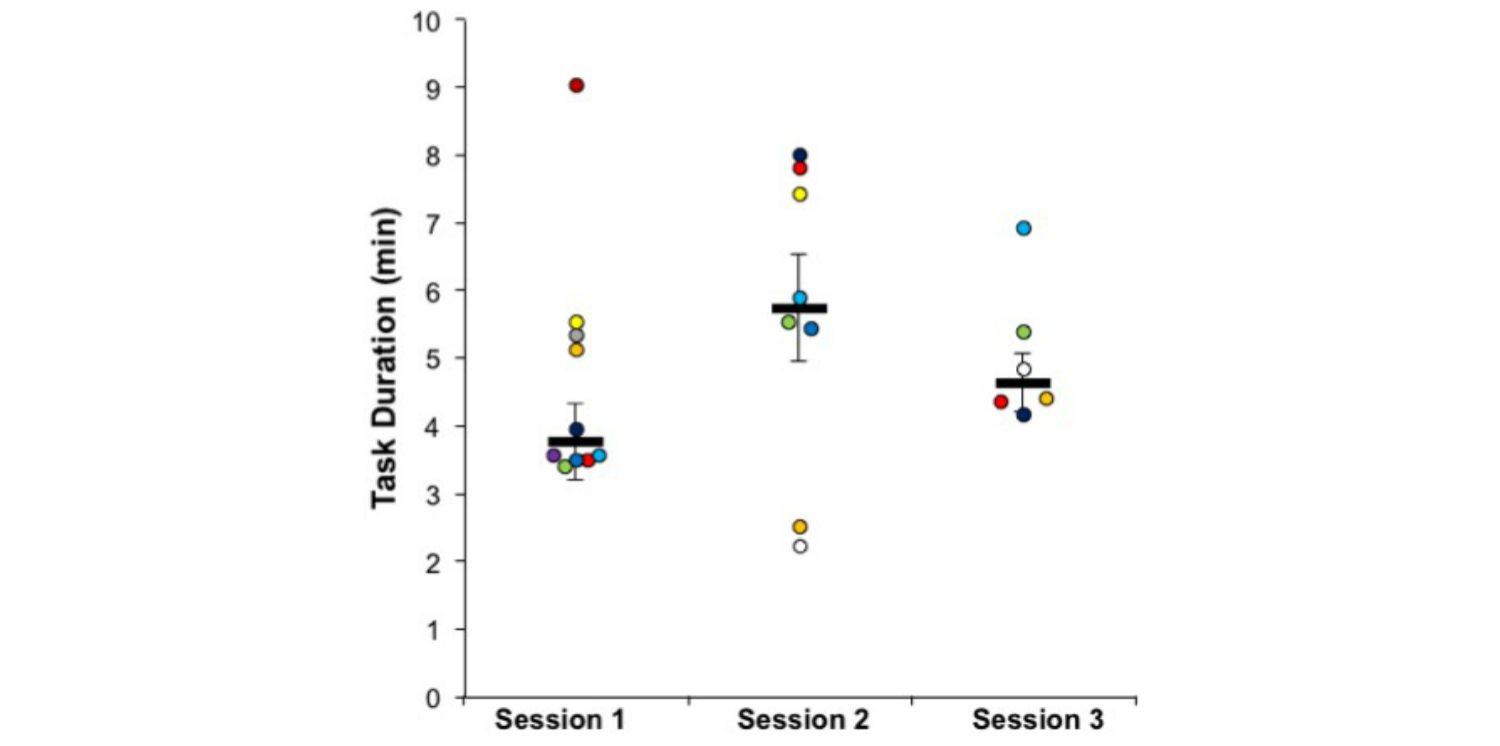
Time taken by each participant in the clinical group to complete the task in each intervention session.

Regarding the task duration, since the tasks increased in complexity and difficulty along the intervention, the task duration is not directly comparable. However, it is important to verify that the time to successfully complete the task did not statistically increase, even with exposure to harder levels (Figure 10). In session 1, the task was performed in the “easy” mode, in which players are guided step by step, from the starting position until the final location. Tasks in “easy” mode do not require as much planning as the ones in “hard” mode, but slowly introduce the player to this concept and demonstrate how buses can be used to travel between bus stations. The “hard” mode tasks, on the other hand, only tell the player where they must go to complete the task (eg, “go to the hospital”). In these tasks, players have to analyze the map to discover which bus or buses they can take to reach the final destination. Furthermore, the tasks received in sessions 1 and 2 required the player to take 1 bus, while the task from session 3 required the player to take 2 buses. A Wilcoxon signed-rank test show no difference between the easiest and simpler (first session) task and the most complex and difficult task (last session), Z=0.105, *P*=.92.

**Figure 11:**
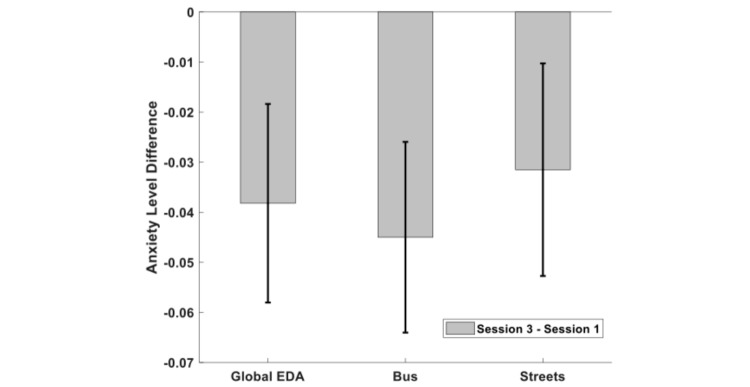
Decrease observed in anxiety levels, measured by electrodermal activity variability, between the last session and the first. EDA: electrodermal activity.

The anxiety levels decreased from the first to the last session, for both overall EDA and for the bus and streets conditions. Figure 11 shows the mean differences and their error. However, only the bus condition shows a strong tendency to significance (t(5)=−2.36, *P*=.07). The general EDA values showed a weak tendency towards an effect (t(5)=−1.93, *P*=.11) and the EDA values on the streets had the smaller decrease with the intervention (t(5)=−1.48, *P*=.20).

**Figure 12:**
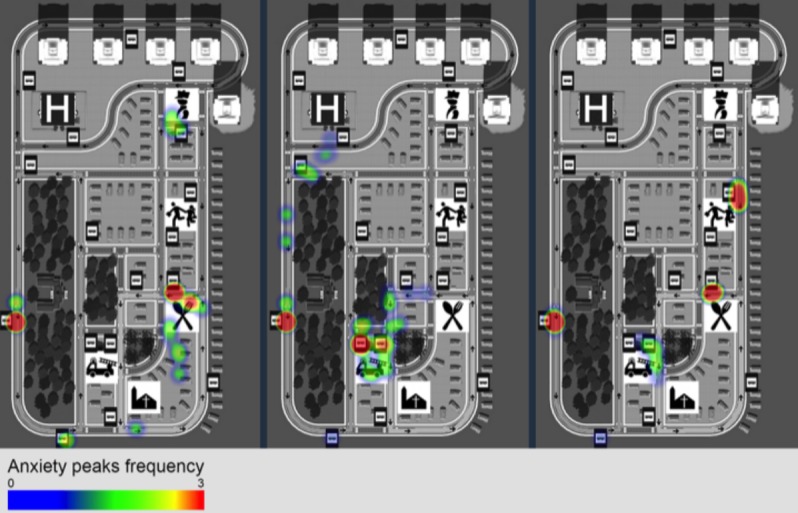
Anxiety peaks heat map from session 1 (left) to session 3 (right) for the times the participant was not inside of the bus environment. Most of the locations represent bus stops, where participants need to make the decision of what bus to take and wait for it to arrive.

**Figure 13:**
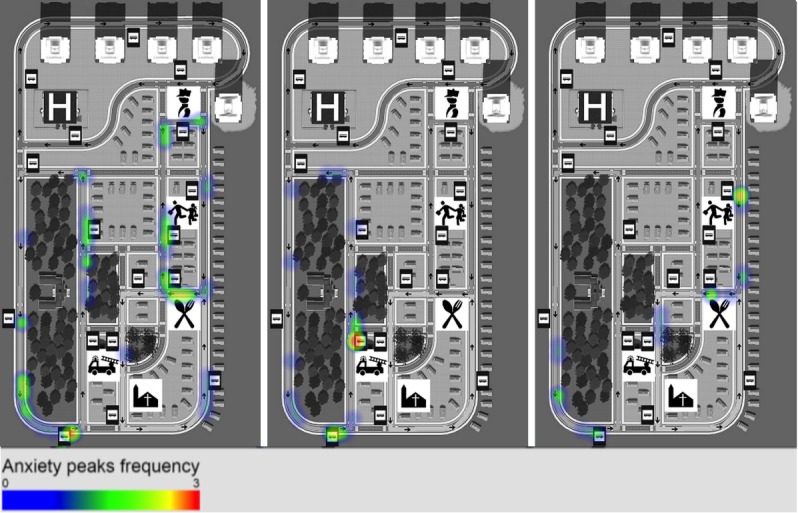
Anxiety peaks heat map from session 1 (left) to session 3 (right) for the times the participant was inside of the bus environment. The locations are much more dispersed through the route than in the outside the bus scenario. There is a visible decrease in frequency of anxiety peaks from the first to the last session.

We created heatmaps using the peaks of anxiety of each participant and separated the conditions inside the bus and outside. Figure 12 shows the streets scenario for each of the sessions, and Figure 13 shows the same metrics but for the inside the bus condition.

According to Figure 12, players felt most anxious in 3 different situations (Textbox 2).

**Situations in which players felt anxious.**

- When waiting for the bus at the bus stops (red areas near the bus stop signs)
- When planning the trip or looking for the correct bus stop in the starting areas (ie, green areas near the restaurant in session 1, the fire station in stage 2, one of the bus stops in session 3)
- When reaching or looking for the destination in the finishing areas (ie, green areas near the police station in session 1, the hospital in session 2, and the fire station in session 3).

## Discussion

### Principal Findings

This project aimed to assess the potential of the developed serious game and here presents it as a tool for rehabilitation. The game is, to our knowledge, the first application specifically developed to teach ASD individuals to use public transportation systems. In addition, we were able to measure psychophysiological markers of anxiety, paving the way for future biofeedback applications. With only three sessions, it was possible to improve the knowledge of the participants regarding the norms of the bus-taking process and to reduce the anxiety levels felt by the participants during that process.

The impacts of a learning tool with this purpose are broad since it trains executive functions and might increase the autonomy of the users, providing them with a new way of moving through a city. It is also a way to make cities more inclusive, providing people with special needs ways to successfully use this type of public service.

Some studies have been conducted using VR training for ASD, usually focusing on training other skills. Most interventional approaches target social performance training [18,19] or job interviewing [20]. Gaming platforms [21] and brain-computer interfaces [22] were also suggested in the literature for autism training, but without validation with patients. Although these are important targets of intervention, our work focuses on a more specific task of executive function that is relevant for the needs of daily life. Our pilot validation study aimed to assess not only the efficacy of the application, but also the acceptance of the solution with this specific clinical population. Few studies perform fully immersive interventions, and the difficulties of combining them with biofeedback create a technology apparatus that could potentially be disruptive to the participants. The 4 drop-outs during the intervention occurred exclusively due to scheduling issues. No patient dropped the study due to discomfort or raised any difficulty in using the setup. There were specific cases where the Oculus device was not used, but all of those were related to vision impairments, not to lack of tolerance from the user.

The baseline comparison with the control group was clear in identifying the impairments in the clinical group. Both the debriefing of the procedure for taking the bus and the in-game actions showed statistically different results between groups, proving the validity of the rehabilitation target and confirming the capacity of the game to, by itself, identify the deficits of target participants in the process of taking the bus. Additionally, the time needed to complete the task was increased for the clinical group. Despite having mean anxiety levels above the control group, this difference was not significant, perhaps due to the small statistical power resulting from a small group of participants, as well as the implementation of biofeedback, which decreases differences between groups.

The intervention was successful in increasing the accuracy of the process description during the debriefing, showing a statistically significant improvement in the theoretical knowledge of the process, which was the main outcome measure. When evaluated inside the game by the user actions, the increase was not statistically significant, but showed a tendency that we believe additional sessions or a larger intervention group would further confirm. It was also successful in decreasing the anxiety felt by participants, especially inside the bus. By using heat maps to represent the anxiety peaks recorded, it was possible to understand that participants with ASD felt more anxious in bus stops and near the starting and finishing areas. This led to the conclusion that, when outside the buses, players felt most anxious when planning the trip, when looking for the bus stop, when waiting for the bus, and when looking for the final destination. Inside the bus, we observed a desensitization to stress throughout the sessions, with a final session showing fewer peaks of EDA activity.

Despite the increase of task complexity and difficulty across sessions, the time duration to complete the task did not increase, suggesting a learning effect and adaptation to the serious game.

### Conclusions

By using the game as a therapeutic intervention tool, in just three sessions it was possible to improve the general efficiency of participants and expose them to peculiar scenarios in which they could train their planning skills. More importantly, it was possible to nearly extinguish the anxiety felt in bus environments and teach the bus-taking norms necessary for the autonomous use of buses for transportation, both in theoretical and practical contexts. Future studies should conduct randomized controlled trials, with larger intervention groups, to replicate the findings and extend them to other clinical populations with executive function deficits and lack of autonomy.

## Abbreviations

ASD: Autism Spectrum Disorder
EDA: electrodermal activity
HMD: head-mounted display
IQ: Intelligence Quotient
TD: typical development
VR: virtual reality
